# Effects of Internet-Based Nutrition and Exercise Interventions on the Prevention and Treatment of Sarcopenia in the Elderly

**DOI:** 10.3390/nu14122458

**Published:** 2022-06-14

**Authors:** Zhengyuan Wang, Xin Xu, Shanxi Gao, Chunxiang Wu, Qi Song, Zehuan Shi, Jin Su, Jiajie Zang

**Affiliations:** 1 Division of Health Risk Factors Monitoring and Control, Shanghai Municipal Center for Disease Control and Prevention, Shanghai 200336, China; wangzhengyuan@scdc.sh.cn (Z.W.); xuxin_0316@126.com (X.X.); songqi@scdc.sh.cn (Q.S.); shizehuan@scdc.sh.cn (Z.S.); sujin@scdc.sh.cn (J.S.); 2 Division of Health Risk Factors Monitoring and Control, Shanghai Municipal Fengxian District Center for Disease Control and Prevention, Shanghai 200336, China; fxcdcshipin@126.com; 3 Division of Chronic Disease Control, Shanghai Municipal Putuo District Center for Disease Control and Prevention, Shanghai 200336, China; wcx8127@163.com

**Keywords:** sarcopenia, nutrition, exercise, application, elderly

## Abstract

Effective nutrition and exercise interventions may improve sarcopenia in the elderly. The purpose of our study was to investigate the effectiveness of Internet-based nutrition and exercise interventions in the elderly with sarcopenia. Participants were divided into 4 groups: control, nutrition, exercise, and comprehensive (nutrition plus exercise) groups; there was at least 50 participants in each group. Our trial lasted 12 weeks. We conducted dietary and exercise interventions through an app and collected feedback from the participants every three weeks. Information on the diet, skeletal muscle mass, and muscle function was collected before and after the interventions. The comprehensive group had higher high-quality protein intake than the control (*p* = 0.017) and exercise (*p* = 0.012) groups. After the interventions, we obtained differences in skeletal muscle mass, skeletal muscle mass/height^2^, skeletal muscle mass/weight, muscle mass/BMI, and skeletal muscle mass/body fat percentage (*p* < 0.05). Changes in average daily energy and total daily protein intakes were not significantly different; however, there was an overall improvement in the intervention groups relative to baseline data. There were no changes in the average daily time of moderate physical activity. The Internet was an effective tool of nutrition intervention in the elderly with sarcopenia. The Internet-based nutrition intervention improved high-quality protein intake and skeletal muscle mass in the elderly with sarcopenia.

## 1. Introduction

According to the World Health Organization (WHO), a country or region enters the advanced aging stage when the proportion of individuals of 65 years of age or over is >14%. In Shanghai, China, the advanced aging stage is significant. Towards the end of 2020, Shanghai’s registered population over 65 was 25.9% of the city’s registered population [1], which is 1.85 times the WHO standard. Typical manifestations of aging include the loss of muscle fiber quality (including volume and quantity), decrease in muscle strength and endurance, and reduction in metabolic capacity [2], which may contribute to the development of sarcopenia, weakness, fractures, and other adverse events including death.

The incidence of sarcopenia has gradually increased in recent years. According to the third National Health and Nutrition Examination Survey, the prevalence of second-degree sarcopenia in men and women over 60 years in the United States was 9.3% and 7.3%, respectively, and the prevalence increased with age [3]. In 2019, the Asian Sarcopenia Working Group reported that the prevalence of sarcopenia in the elderly population in Asia ranged from 5.5% to 25.7% [4]. A community-based study in Shanghai showed that the prevalence of sarcopenia in men and women over 60 was 14.9% and 14.0%, respectively [5].

There are no suitable clinical interventions for sarcopenia. Nutrition and exercise are the primary methods to prevent and treat sarcopenia. Specifically, adequate intakes of energy, protein, especially high-quality protein [6], long-chain polyunsaturated fatty acids [7], amino acids, especially branched-chain amino acid [8], vitamin D [9], and antioxidants [10] reduce the decline in skeletal muscle mass and muscle strength among the elderly. Aerobics [11], endurance exercise [12], and resistance training [13] significantly increase muscle mass and strength in patients with sarcopenia.

The Consensus of Chinese Experts on Nutrition and Exercise Intervention for Muscle Attenuation Syndrome in 2015 [14] provides nutrition and exercise programs for sarcopenia treatment. The nutrition program mainly includes the recommended protein intake of 1.0~1.5 g/kg, of which the proportion of high-quality protein better reach 50%, and vitamin D supplementation of 600~800 IU/d. The exercise program mainly consists of a recommended total of 40–60 min of moderate-to-high-intensity exercise per day. However, nutrition and exercise interventions require dynamic adjustments, and the interventions of this expert consensus need to be guided by professionals to benefit patients with sarcopenia, which is not suitable for large-scale community interventions. The Internet is an optimal venue for healthcare information. Compared with traditional face-to-face interventions, the Internet can save manpower and material resources and does not limit time or space [15,16].

Our study used an app to provide health education and personalized guidance to the elderly with sarcopenia based on the Consensus of Chinese Experts released in 2015. The purpose of our study was to explore the effectiveness of Internet-based nutrition and exercise interventions.

## 2. Materials and Methods

### 2.1. Study Sample

We used the formula of randomized controlled trials to calculate the sample size: n = 2σ2 × f(α,β)/(μ1 − μ2)2, where α = 0.05, β = 0.20, and f(α,β) = 7.9. Based on research data, the standard deviation of skeletal muscle was σ = 2.25, μ1 − μ2 = 1.27 [17]. At least 50 participants in each group should complete the trial. Considering the loss to follow-up, we recruited n/0.8 (62) participants for each group.

Our study included a control group and three intervention groups: nutrition, exercise, and comprehensive (nutrition plus exercise) groups. For field feasibility, we randomly selected 4 districts from 16 districts in Shanghai, with a community healthcare center chosen at random from each project area as the test site. The control group originated from Jinhai Community Health Service Center in Fengxian District, all participants in this community were included in this group, and participants in other communities were enrolled in the same manner. The nutrition, exercise, and comprehensive groups originated from the Ganquan Street Community Health Service Center in Putuo District, the Ying Pu Street Community Health Service Center in Qingpu District, and the Laoximen Community Health Service Center in Huangpu District, respectively.

Participants (65 to 75 y) had sarcopenia, were able to walk independently, and used a smartphone for 3+ years or were proficient in using a smartphone. Exclusion criteria included the presence of cognitive disorders; infectious diseases or diseases that affected the heart, liver, or kidneys; hip and knee joint pain or dysfunction; recent trauma, fracture, or surgeries, as well as diseases that prevented exercise. Furthermore, subjects were excluded if they consumed specific drugs that might affect the study outcomes, for example, thyroid hormones, steroids, weight-loss drugs, etc.

Our study started in November 2020 and ended in February 2021 (12 weeks).

The study was conducted in accordance with the Declaration of Helsinki, and the protocol was approved by the Shanghai Municipal Center for Disease Control and Prevention Ethical Review Committee (2019-46). All of the participants provided informed consent. The study protocol was registered in the Chinese Clinical Trial Registry (ChiCTR2100048874).

### 2.2. Sarcopenia Criteria

To assess sarcopenia, we used a combination of clinical diagnostic methods from the consensus updated by the European Working Group on Sarcopenia in Older People (EWGSOP) in 2018 and criteria from the consensus updated by the Asian Working Group on Sarcopenia (AWGS) in 2019 [4,18].

**Figure 1:**
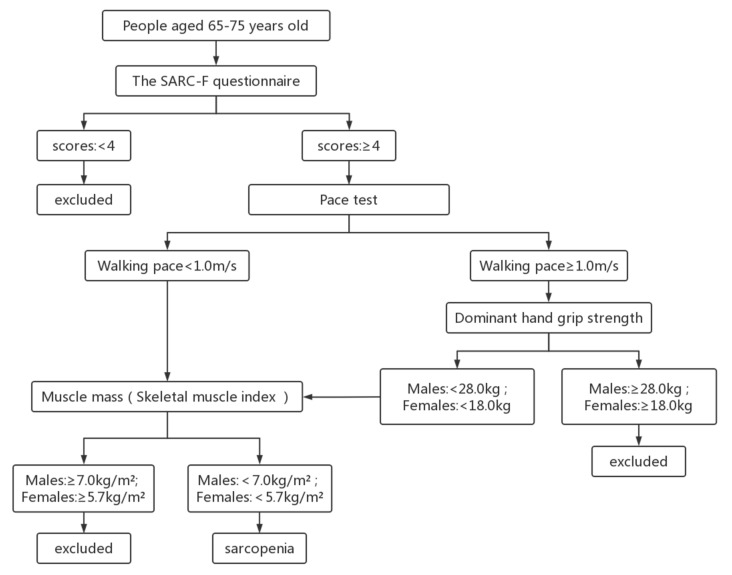
Assessment and screening process for sarcopenia.

The SARC-F questionnaire recommended by EWGSOP was used to screen the elderly in the community. Participants with scores ≥ 4 were further examined as recommended by AWGS (Figure 1).

### 2.3. Interventions

After screening and baseline surveys, we informed the participants of their disease status and provided them with health education on sarcopenia, including the role of nutrition and exercise in sarcopenia, but not how nutrition and exercise adjustments were made. The control group only received health education without any intervention. At the same time, participants in the three intervention groups were trained in the use of the application (APP), including dietary or exercise assessments, feedback, and recommendations for improvement. The nutrition group received dietary management information through an app. The app featured the ability to assess each participant’s diet and provide recommendations for adjustments, focusing on energy and protein intake, especially the high-quality protein, and give recommended recipes. For example, the APP would show that the gap between the recommended intake and actual intake of energy and protein and every 50 g of fish and shrimp or 25 g of lean meat or 40 g of poultry contains 10 g of high-quality protein, etc., which was convenient for participants to adjust their intake spontaneously. The exercise group received exercise management information. The app evaluated the participant’s exercise status and recommended the amount of exercise, such as 40 to 60 min of moderate-to-high-intensity exercise (brisk walking and jogging) and resistance training (seated leg raises, static squat against a wall, dumbbell lifts, elastic bands, etc.) for 30 min, 3 days a week or more. The comprehensive group received both dietary and exercise management information. The specific content was consistent with the above two intervention groups. Every 2 weeks, the app’s automatic reminder function would remind participants for assessment and feedback. At least five assessments and feedback had to be completed. After 12 weeks, all participants had free access to the full version of the app.

Our team did not actively contact the participants during the intervention period, unless the participants had difficulty on using the APP, and this contact did not include any nutritional or exercise instructions.

### 2.4. Data Collection

The survey contents were consistent before and after the intervention including questionnaires, physical examinations, and muscle function tests.

Questionnaires consisted of a general situation questionnaire (including age, gender, education level, marital status, pre-retirement occupation, and smoking status), food frequency questionnaire (FFQ), disease history survey, and activity quantitative survey. The FFQ was a validated and reliable questionnaire including 15 food categories with a total of 117-item to assess the frequency and average intake of food in the past three months [19]. These food categories included: cereals (including noodles, maize potatoes and rice), soyabean, vegetables, fruit, fungus and algae, milk and dairy products, meat and poultry, fish and seafood, eggs, preserved food, alcohol, beverages, snacks, oil and condiments, dietary supplement.

High-quality protein was defined as protein derived from soyabean, milk and dairy products, meat and poultry, fish and seafood, eggs, preserved animal food, snacks, dietary supplement.

We used calibrated electronic grip actuator (CAMRY, EH101, range 0–90 kg, indexing value 0.1 kg) to assess grip strength and INBODY 770 (Biospace, Seoul, Korea) to assess height, waist circumference, hip circumference, and blood pressure.

Muscle function tests included balance test, 4-m timed walking test, and timing sitting and standing tests.

The primary outcomes were changes in skeletal muscle mass, the other indicators include muscle function (including balance test, timed 4-m walk test, and timed sit-up test).

### 2.5. Statistical Analysis

We used IBM SPSS Statistics version 21.0 (IBM Corp., Armonk, NY, USA) to analyze the data. Normally distributed data were presented as mean ± SD (standard deviation). Factors measured at baseline such as gender, age, education level, marital status, pre-retirement occupation, smoking, energy intake, total protein intake, high-quality protein intake and BMI etc. that may influence the outcome of the intervention were analyzed by propensity score method. The score was used as covariate in analysis of covariance of multi-group comparisons. We used one-way analysis of variance (ANOVA) for multi-group comparisons. The test for homogeneity of variance was >0.05. LSD was used for post-hoc pairwise comparison and when it was <0.05, we used Tamhane’s T2 for comparisons. Classification data were expressed by composition ratio, and the differences of each group were compared by Chi-square test; *p* < 0.05 was statistically significant.

## 3. Results

### 3.1. Study Population and Use Frequency of APP by Participants

There were 54, 58, 62, and 60 participants in the control, nutrition, exercise, and comprehensive groups, respectively. The number of participants who completed the 12-week follow-up were 51 (control group), 50 (nutrition group), 50 (exercise group), and 50 (comprehensive group), which met the minimum sample size required for the study. The lost to follow-up rates were 5.6% (control group), 12.1% (nutrition group), 19.4% (exercise group), and 16.7% (comprehensive group).

The frequency of app feedback/evaluation provided by the different intervention groups had no statistically significant differences (Table 1).

### 3.2. Baseline Comparisons

There were significant differences in age, educational level, marital status, pre-retirement occupation, and skeletal muscle mass among the 4 groups (*p* < 0.05). Based on age and skeletal muscle mass, the nutrition group was younger than the other three groups (control group vs. nutrition group, *p* = 0.026; comprehensive group vs. nutrition group, *p* = 0.010; exercise group vs. nutrition group, *p* = 0.044). Skeletal muscle mass was higher in the comprehensive group than in the control (*p* = 0.006) and nutrition (*p* = 0.031) groups. There were no significant differences among the groups in gender, smoking, energy, total protein, high-quality protein, or BMI (Table 2).

### 3.3. Comparison of Diet and Exercise Status before and after the Intervention

There were no significant differences among the four groups in energy intake, protein intake, and moderate physical activity. However, there were significant differences among the groups in the daily average intake of high-quality protein and BMI (*p* < 0.05). Specifically, daily average intake of high-quality protein was higher in the comprehensive group than in the control and exercise groups (*p*1 = 0.017, *p*5 = 0.012) and BMI was the highest in the nutrition group followed by the comprehensive, control, and exercise groups (*p*1 = 0.006, *p*2 = 0.022, *p*5 < 0.001, *p*6 < 0.001; Table 3).

### 3.4. Comparison of Quantity and Function of Skeletal Muscle before and after the Intervention

We compared the changes in skeletal muscle mass and related indexes of muscle function among the groups before and after the intervention. There were no significant differences among the 4 groups before and after the intervention in the changes in balance test (the third posture), 4-m regular walking time, and regular sitting and standing times. The changes in skeletal muscle mass, skeletal muscle mass/height^2^ in female, skeletal muscle mass/body weight, skeletal muscle mass/BMI, skeletal muscle mass/body fat percentage were significant among the 4 groups before and after the intervention (*p* < 0.05).

Pairwise analysis showed that skeletal muscle mass after the intervention was higher in the comprehensive and nutrition groups than in the control and exercise groups (*p*1, *p*2, *p*5 < 0.001, *p*6 = 0.004). Skeletal muscle mass/height^2^ in female was higher in the comprehensive and nutrition groups than in the control and exercise groups (*p*1, *p*2, *p*5 < 0.001, *p*6 = 0.003). Compared to the control group, the comprehensive, nutrition, and exercise groups had higher skeletal muscle mass/body weight (*p*1 = 0.001, *p*2 = 0.040, *p*3 = 0.002). Skeletal muscle mass/BMI was higher in the comprehensive and exercise groups than in the control group (*p*1 = 0.001, *p*3 = 0.006). Finally, skeletal muscle mass/body fat percentage was higher in the comprehensive, nutrition, and exercise groups than in the control group (*p*1 = 0.011, *p*3 = 0.013; Table 4).

**Table 3:** Comparison of nutrient intake and exercise status before and after the intervention.

Variable	Control Group	Comprehensive Group	Nutrition Group	Exercise Group	*p*	*p*1	*p*2	*p*3	*p*4	*p*5	*p*6
Energy, kJ					0.118	0.061	0.034	0.364	0.594	0.293	0.154
Baseline	7266 ± 3441	7204 ± 2789	7704 ± 3773	7711 ± 3914							
After the intervention	7362 ± 3081	8857 ± 3844	9154 ± 2744	8343 ± 4176							
Total protein, g					0.081	0.059	0.124	0.930	0.924	0.038	0.094
Baseline	70.29 ± 39.24	69.10 ± 23.12	83.07 ± 41.70	73.06 ± 41.09							
After the intervention	78.08 ± 45.47	92.28 ± 45.67	97.25 ± 34.59	76.74 ± 36.07							
High-quality protein, g					0.022	0.017	0.076	0.990	0.778	0.012	0.067
Baseline	37.98 ± 27.66	37.72 ± 12.10	45.25 ± 27.56	38.49 ± 32.31							
After the intervention	36.26 ± 15.89	51.25 ± 26.54	54.62 ± 23.20	38.28 ± 20.05							
BMI, kg/m^2^					<0.001	0.006	0.022	0.080	0.943	<0.001	<0.001
Baseline	22.67 ± 3.07	23.68 ± 3.87	22.26 ± 2.4	23.15 ± 2.89							
After the intervention	23.35 ± 3.14	25.30 ± 3.70	24.60 ± 3.22	23.26 ± 2.82							
Average daily time of moderate physical activity, median (P25, P75)					0.759						
Baseline	0.00 (0.00, 14.28)	0.00 (0.00, 0.00)	0.00 (0.00, 17.68)	0.00 (0.00, 37.50)							
After the intervention	0.00 (0.00, 0.00)	0.00 (0.00, 2.89)	0.00 (0.00, 25.71)	10.00 (0.00, 45.00)							

*p*: Comparison between 4 groups of difference values; *p*1: control group vs. comprehensive group; *p*2: control group vs. nutrition group; *p*3: control group vs. exercise group; *p*4: comprehensive group vs. nutrition group; *p*5: comprehensive group vs. exercise group; *p*6: nutrition group vs. exercise group.

## 4. Discussion

The rapidly growing elderly population has led to an increased incidence of several degenerative diseases. Sarcopenia, a common degenerative disease, is of public concern and contributes to a considerable medical burden. In 2000, medical expenses from sarcopenia treatment were ~1.85 billion dollars in the United States [20]. Due to China’s vast population and rapid aging, the healthcare burden from sarcopenia will be greater than that in the United States. However, sarcopenia, which is not linked to hospitalization [21,22], can be alleviated with appropriate nutrition and exercise interventions under the guidance of a trained specialist. Our study findings revealed that an Internet-based nutrition intervention through an app improves high-quality dietary protein intake, BMI, and skeletal muscle mass in the elderly.

According to our findings, the elderly are willing to use the Internet for self-treatment. More than 87% of the participants completed 3 or more assessments on their own probably as a result of the growing number of Internet-based home management tools, such as smart wearable devices, application software for health management, and online doctor-patient communication platforms [23]. Therefore, the elderly are not unfamiliar with apps. A recent systematic revealed that older adults tend to use the Internet to search more disease-related health information than the adolescent and youth population [24]. Our study provided an app as a guiding tool for the elderly with sarcopenia to acquire accurate information in a convenient and low-cost approach [25]. The findings revealed that apps are effective, particularly for nutrition interventions. Participants formed good nutrition habits through the guidance of the app, as seen by the significant increase in their high-quality protein intake and BMI.

High-quality protein intake increased by ~35% in the comprehensive group. A systematic review and meta-analysis that included 14 studies and 1424 participants showed that the intake of a variety of high-quality dietary proteins increases muscle anabolism in older adults [26]. High-quality proteins such as whey protein, soy protein, and the soy/whey protein combinations are associated with muscle anabolism [13,27,28]. In addition, studies have pointed out that, regardless of the source of dietary protein, increased total protein intake may also be beneficial for sarcopenia. The PROT-AGE Study Group reported that daily protein intakes between 1.2 and 1.5 g of protein per kg of body weight (g/kg/day) may be more beneficial for the elderly with chronic diseases than the recommended dietary protein intake (0.8 g/kg/day) [29]. Even though there were no significant differences in the changes in total protein intake before and after interventions, the comprehensive and nutrition groups had higher total protein intake (+33% and +17%, respectively). The optimization and adjustment of the participants’ diet may benefit from the recommended recipes of the APP. It is recommended to use more recipes with quantity or picture descriptions in nutrition education for the elderly, which can help the elderly to have a concept of food share and facilitate their actual operation.

BMI changes were 2× higher in the comprehensive and nutrition groups than in the exercise and control groups. Sarcopenia can be prevented by maintaining a healthy weight or being slightly overweight [30]. According to a cross-sectional survey conducted in Shanghai, individuals with higher BMI may have a higher energy and protein intake, which is a protective factor against muscle loss [31], and our findings supported this. Even though there were no statistically significant differences in the changes inr energy before and after the intervention, there was an increase in energy intake in each group compared to baseline levels. The energy intake of the three intervention groups increased by more than 600 kJ. The energy intake of the comprehensive group increased by 1653 kJ.

Skeletal muscle mass and skeletal muscle mass/height^2^ increased more in the comprehensive and nutrition groups than in the control and exercise groups, consistent with a past study [32]. In the absence of increased physical activity in the elderly, these results may be related to the nutrition intervention, which enhances the dietary habits and physical function of patients with sarcopenia [33]. The skeletal muscle mass of the comprehensive and nutrition groups increased by 1.09 kg and 1.25 kg, respectively, which is higher than that reported by Kang et al. (0.55 kg and 0.71 kg). This result may be related to the different age distribution of the two study groups, which is approximately three years younger in our study than in the Kang et al. study.

Unfortunately, no significant improvement in physical activity was observed in the exercise intervention group and the comprehensive intervention group. And the elderly did not participate in the recommended exercises, which could explain why we did not observe any changes in muscle function. Changing the exercise habits of older adults can be relatively difficult. A national study conducted in the United Kingdom found that, while the elderly expressed a willingness to maintain their exercise routines, many of them did not [34]. Apps can only provide advice and may not be a strong incentive for the elderly to change their physical activity. In addition, according to a randomized controlled experiment conducted in the Netherlands [35], insufficient infrastructure, low self-efficacy, and lack of social support may all pose difficulties for the elderly to change their exercise habits. It is possible that exercise management in older adults still requires the support and companionship of professional. Nonetheless, we insist on encouraging older adults to increase their physical activity. A randomized controlled trial in Germany found that resistance exercise of the lower limbs can effectively enhance skeletal muscle content and improve balance [36], indicating that exercise remains the most helpful strategy to prevent sarcopenia-induced degradation of muscle function. If APP exercise management continues to be implemented in the future, it may require the support and efforts of families, society and government policies to create an environment that facilitates physical exercise for the elderly, pay attention to their ideas, and solve obstacles.

Although the score of propensity score differed among groups, the results were consistent before and after the adjustment; overall, they had little effect on our findings. In our study, the intervention plans were based on the Consensus of Chinese Experts released in 2015, making them more appropriate for the Chinese population and providing targeted interventions. The app provided individualized health guidance to the elderly to accomplish the effect of self-management, and the feedback system allowed us to see each participant’s completion, potentially saving a lot of manpower and material resources.

Our study had some limitations. Due to community-organized considerations, we did not use the gold standard dual energy X-ray absorptiometry to assess sarcopenia. Instead, we used an internationally recognized alternative, bioimpedance analysis (BIA) [37]. In addition, the main evaluation index (skeletal muscle mass) in the baseline characteristics was statistically different among the four groups, and the results obtained may be biased. Moreover, 12.7% of the participants completed only 1 or 2 feedbacks, which might also impact the effectiveness of interventions based on APP.

## 5. Conclusions

The Internet is an effective health tool for elderly patients with sarcopenia. Internet-based nutrition interventions were effective, and they can optimize the diets of elderly patients with sarcopenia and improve skeletal muscle mass; however, they cannot improve skeletal muscle function. The Internet-based exercise intervention have failed by not improving physical activity in the elderly. We recommend the use of the Internet coupled with dietary interventions.

## Figures and Tables

**Table 1:** Frequency of app feedback/evaluation provided by the different intervention groups.

App Evaluation Feedback Completed Times	Comprehensive Group (n)	Nutrition Group (n)	Exercise Group (n)	X^2^	*p*
5 times and above	24	30	25	1.967	0.742
3 to 4 times	20	14	18		
1 to 2 times	6	6	7		

**Table 2:** Basic information of participants in each group.

Variable	Control Group	Comprehensive Group	Nutrition Group	Exercise Group	F/X^2^	*p*
Gender, n (%)					0.779	0.854
Male	7 (13.7)	8 (16.0)	9 (18.0)	10 (20.0)		
Women	44 (86.3)	42 (84.0)	41 (82.0)	40 (80.0)		
Age, y	69.88 ± 3.29	70.16 ± 4.32	68.18 ± 3.93	69.72 ± 3.60	2.740	0.044
Education level, n (%)					77.518	<0.001
High school and above	0 (0)	14 (28.0)	21 (42.0)	4 (8.0)		
Junior high school	10 (19.6)	24 (48.0)	26 (52.0)	16 (32.0)		
Primary school and illiteracy	41 (80.4)	12 (24.0)	3 (6.0)	30 (60.0)		
Marital status, n (%)					9.979	0.019
In marriage	47 (92.2)	36 (72.0)	39(78.0)	45 (90.0)		
Divorced or widowed	4 (7.8)	14 (28.0)	11(22.0)	5 (10.0)		
Pre-retirement occupation, n (%)					58.302	<0.001
Mental work mainly	7 (13.7)	22 (44.0)	44 (88.0)	19 (38.0)		
Physical labor mainly	44 (86.3)	28 (56.0)	6 (12.0)	31 (62.0)		
Smoking, n (%)	2 (3.9)	6 (12.0)	6 (12.0)	8 (16.0)	6.807	0.339
Energy, kj	7266 ± 3441	7204 ± 2789	7704 ± 3773	7711 ± 3914	0.306	0.821
Total protein, g	70.29 ± 39.24	69.10 ± 23.12	83.07 ± 41.70	73.06 ± 41.09	1.466	0.225
High-quality protein, g	37.98 ± 27.66	37.72 ± 12.10	45.25 ± 27.56	38.49 ± 32.31	0.924	0.430
BMI, kg/m^2^	22.67 ± 3.07	23.68 ± 3.87	22.26 ± 2.4	23.15 ± 2.89	1.947	0.123
Skeletal muscle mass, kg	13.98 ± 2.37	15.33 ± 2.95	14.27 ± 2.23	14.37 ± 2.20	2.850	0.039
The score of propensity score	0.59 ± 0.18	0.56 ± 0.18	0.34 ± 0.15	0.49 ± 0.19	19.063	<0.001

**Table 4:** Comparison of quantity and function of skeletal muscle before and after the intervention.

Variable	Control Group	Comprehensive Group	Nutrition Group	Exercise Group	*p*	*p*1	*p*2	*p*3	*p*4	*p*5	*p*6
Skeletal muscle mass, kg					<0.001	<0.001	<0.001	0.243	0.158	<0.001	0.004
Baseline	13.98 ± 2.37	15.33 ± 2.95	14.27 ± 2.23	14.37 ± 2.20							
After the intervention	13.88 ± 2.39	16.42 ± 2.98	15.52 ± 2.39	14.44 ± 2.28							
Skeletal muscle mass/height^2^ in male, kg/m^2^					0.109	0.115	0.062	0.775	0.676	0.115	0.056
Baseline	5.64 ± 0.62	6.23 ± 1.08	5.64 ± 0.54	5.79 ± 0.51							
After the intervention	5.59 ± 0.61	6.64 ± 1.05	6.14 ± 0.70	5.81 ± 0.53							
Skeletal muscle mass/height^2^ in female, kg/m^2^					<0.001	<0.001	0.001	0.263	0.320	<0.001	0.018
Baseline	6.11 ± 0.61	6.88 ± 1.11	6.13 ± 0.68	6.33 ± 0.32							
After the intervention	6.05 ± 0.52	7.37 ± 1.04	6.87 ± 0.74	6.38 ± 0.39							
Skeletal muscle mass/body weight					0.005	0.001	0.040	0.002	0.485	0.874	0.562
Baseline	0.25 ± 0.03	0.26 ± 0.03	0.25 ± 0.03	0.25 ± 0.03							
After the intervention	0.24 ± 0.03	0.26 ± 0.03	0.25 ± 0.03	0.25 ± 0.03							
Skeletal muscle mass/BMI					0.005	0.001	0.077	0.006	0.233	0.425	0.583
Baseline	0.62 ± 0.11	0.65 ± 0.10	0.64 ± 0.10	0.63 ± 0.11							
After the intervention	0.60 ± 0.11	0.65 ± 0.10	0.64 ± 0.10	0.63 ± 0.12							
Skeletal muscle mass/body fat percentage					0.040	0.011	0.070	0.013	0.712	0.894	0.792
Baseline	0.95 ± 0.43	0.83 ± 0.27	0.76 ± 0.23	0.84 ± 0.30							
After the intervention	0.89 ± 0.40	0.85 ± 0.29	0.78 ± 0.26	0.88 ± 0.38							
Balance test (third posture)					0.679	0.427	0.233	0.527	0.598	0.851	0.487
Baseline	1.69 ± 0.58	1.62 ± 0.70	1.78 ± 0.51	1.72 ± 0.45							
After the intervention	1.52 ± 0.50	1.66 ± 0.66	1.82 ± 0.48	1.74 ± 0.44							
4-m timed walking test					0.732	0.636	0.269	0.483	0.475	0.825	0.586
Baseline	5.12 ± 1.21	5.19 ± 2.09	5.08 ± 1.15	5.01 ± 1.29							
After the intervention	5.01 ± 1.83	5.13 ± 2.38	5.06 ± 1.16	4.94 ± 1.01							
Timing sitting and standing test					0.942	0.549	0.871	0.849	0.722	0.662	0.996
Baseline	10.71 ± 3.41	11.31 ± 5.63	11.73 ± 4.57	11.66 ± 4.02							
After the intervention	10.72 ± 4.34	9.82 ± 4.54	12.02 ± 3.44	11.51 ± 4.44							

*p*: Comparison among 4 groups of difference values; *p*1: control group vs. comprehensive group; *p*2: control group vs. nutrition group; *p*3: control group vs. exercise group; *p*4: comprehensive group vs. nutrition group; *p*5: comprehensive group vs. exercise group; *p*6: nutrition group vs. exercise group.
